# Population diversity and virulence characteristics of *Cryptococcus neoformans/C*. *gattii* species complexes isolated during the pre-HIV-pandemic era

**DOI:** 10.1371/journal.pntd.0008651

**Published:** 2020-10-05

**Authors:** Sujiraphong Pharkjaksu, Kyung J. Kwon-Chung, John E. Bennett, Popchai Ngamskulrungroj

**Affiliations:** 1 Department of Microbiology, Faculty of Medicine Siriraj Hospital, Mahidol University, Bangkok Noi, Bangkok, Thailand; 2 Laboratory of Clinical Immunology and Microbiology, National Institute of Allergy and Infectious Diseases, National Institutes of Health, Bethesda, Maryland, United States of America; Duke Global Health Institute, UNITED STATES

## Abstract

Cryptococcosis has become a major global health problem since the advent of the HIV pandemic in 1980s. Although its molecular epidemiology is well-defined, using isolates recovered since then, no pre-HIV-pandemic era epidemiological data exist. We conducted a molecular epidemiological study using 228 isolates of the *C*. *neoformans/C*. *gattii* species complexes isolated before 1975. Genotypes were determined by *URA5* restriction fragment length polymorphism analysis and multi-locus sequence typing. Population genetics were defined by nucleotide diversity measurements, neutrality tests, and recombination analysis. Growth at 37°C, melanin synthesis, capsule production, and urease activity as virulence factors were quantified. The pre-HIV-pandemic isolates consisted of 186 (81.5%) clinical, 35 (15.4%) environmental, and 7 (3.1%) veterinary isolates. Of those, 204 (89.5%) belonged to *C*. *neoformans* VNI (64.0%), VNII (14.9%) and VNIV (10.5%) while 24 (10.5%) belonged to *C*. *gattii* VGIII (7.5%), VGI (2.6%) and VGII (0.5%). Among the 47 sequence types (STs) identified, one of VNII and 8 of VNIV were novel. ST5/VNI (23.0%) in *C*. *neoformans* and ST75/VGIII (25.0%) in *C*. *gattii* were the most common STs in both species complexes. Among *C*. *neoformans*, VNIV had the highest genetic diversity (Hd = 0.926) and the minimum recombination events (Rm = 10), and clinical isolates had less genetic diversity (Hd = 0.866) than environmental (Hd = 0.889) and veterinary isolates (Hd = 0.900). Among *C*. *gattii*, VGI had a higher nucleotide diversity (π = 0.01436) than in VGIII (π = 0.00328). The high-virulence genotypes (ST5/VNI and VGIIIa/serotype B) did not produce higher virulence factors levels than other genotypes. Overall, high genetic variability and recombination rates were found for the pre-HIV-pandemic era among strains of the *C*. *neoformans/C*. *gattii* species complexes. Whole genome analysis and *in vivo* virulence studies would clarify the evolution of the genetic diversity and/or virulence of isolates of the *C*. *neoformans/C*. *gattii* species complexes during the pre- and post-HIV-pandemic eras.

## Author summary

Since the beginning of the HIV pandemic in 1980, infections due to isolates of the *Cryptococcus neoformans/C*. *gattii* species complexes have caused many deaths worldwide, especially in the HIV-infected population. Annually, approximately one-third, of all AIDS-related deaths,—representing more than 1,000,000 cases,—are caused by cryptococcosis. Since 1980, extensive molecular epidemiological surveys have been conducted, and the VNI molecular type has been found to be responsible for more than 90% of cryptococcosis in HIV patients. Whether the high VNI prevalence is associated with the HIV pandemic remains controversial as information on the isolates of the pre-HIV pandemic era is lacking. Therefore, this study of the molecular epidemiology and *in vitro* characteristics of the strains from the pre-HIV-pandemic era was undertaken. We found that only 64% of cryptococcosis was caused by VNI, and 9 sequence types existed only in the pre-HIV pandemic era. Unlike what was already known about the strains collected during the HIV pandemic era, ST5 and VGIIIa,—supposedly high virulence genotypes,—did not express higher virulence factors than other genotypes. These results implied that the HIV pandemic altered both the molecular epidemiology and virulence of *Cryptococcus neoformans/C*. *gattii* species complexes have been altered during HIV pandemic. However, detailed mechanism of these alteration remains to be deciphered further.

## Introduction

*C*. *neoformans* and *C*. *gattii* species complexes are the etiologic agents of human and animal cryptococcosis. *C*. *neoformans* is known to infect mainly immunocompromised hosts, whereas *C*. *gattii* infects previously healthy individuals more often than those with known immunosuppression [1, 2]. Cryptococcosis is initiated by the inhalation of infectious propagules (basidiospores or dehydrated yeasts), which colonize the lungs and hematogenously disseminate to the central nervous system [3]. PCR-fingerprinting, restriction fragment length polymorphism analysis of the orotidine monophosphate pyrophosphorylase gene (*URA5*), multi-locus microsatellite typing (MLMT), and multi-locus sequence typing (MLST) analysis have been used to classify *C*. *neoformans* and *C*. *gattii* into eight major molecular types. They are *C*. *neoformans* VNI (var. *grubii*, serotype A); VNII (var. *grubii*, serotype A); VNIII (serotype AD); VNIV (var. *neoformans*, serotype D) and *C*. *gattii*, VGI (serotype B); VGII (serotype B); VGIII (serotype B and C); and VGIV (*C*. *gattii*, serotypes B and C). Among the global isolates from clinical, veterinary, and environmental sources, the molecular types VNI and VGII have been the most common ones identified [4–6]. MLST has become the most widely employed method for cryptococcal molecular epidemiology due to its high discriminatory power and reproducibility as well as the availability of a large online database which allows accurate interlaboratory comparisons between isolates collected world-wide [7, 8]. Studies on the genetic structure of *C*. *neoformans* VNI and VNII using MLST in Asia, Europe, South Africa, and South America have revealed that the majority of isolates belong to sequence types (STs) 4, 5, 6, 23, 63 and 93 [9–12].

It remains unclear whether the rise in the prevalence of cryptococcosis following the advent of the HIV pandemic in the 1980s was caused solely by the deficient immune status of AIDS patients or in combination with the evolution of highly virulent cryptococcal strains. *C*. *neoformans* ST5/VNI and *C*. *gattii* ST20/VGIIa were proposed to be high-virulence genotypes causing outbreaks in many countries [13–17]. Moreover, although *C*. *gattii* was previously thought to be mainly an opportunistic pathogen, VGIII infections have been increasingly reported among immunocompetent patients and animals in North and South America [18–21]. A recent study showed that VGIIIa (VGIII/serotype B) was more virulent in mice than its counterpart, VGIIIb (VGIII/ serotype C) [18, 22]. It has been speculated that natural evolution had resulted in the emergence of virulence difference between the two VGIII subgroups. Extensive molecular epidemiological studies using isolates from various regions around the world have been conducted to verify the evolution of different traits among molecular types [4, 23–25].

However, no data obtained by using sequencing-based methods are available on the molecular epidemiology and population structure of the *C*. *neoformans/C*. *gattii* species complexes isolates in the pre-HIV pandemic era. With this in mind, we investigated the genetic diversity and *in vitro* virulence factors of the *C*. *neoformans/C*. *gattii* species complexes in 228 clinical, environmental, and veterinary isolates recovered during the pre-HIV-pandemic era.

## Materials and methods

### Study isolates

A total of 228 cryptococcal strains from the pre-HIV-pandemic era were obtained from the National Institutes of Health in Bethesda, Maryland and were maintained in a 20% glycerol stock (-80°C) at Siriraj Hospital, Mahidol University. Each isolate was removed from glycerol stock and cultured on Sabouraud dextrose agar (4% dextrose, 1% peptone, 1.5% agar, and final pH 5.6 ± 0.2; Oxoid Ltd, Basingstoke, UK) at 30°C for 48–72 hours, and its species status was confirmed with the RapID Yeast Plus System (Thermo Fisher Scientific, Waltham, MA, USA). *URA5* restriction fragment length polymorphism was performed to differentiate between *C*. *neoformans* and *C*. *gattii*. This study was carried out with the prior approval of the Siriraj Institutional Ethics Committee (Si 091/2016). Only one representative strain per patient or source was used for all genetic diversity analyses. For patient B, all sequential strains were used for the genetic diversity analysis as the sequential strains of this patient belonged to different ST (S1 Table).

### Genotype and MLST analysis

DNA was extracted using chemical lysis solutions with heating according to a previous protocol, but with minor modifications [26]. The *URA5* gene was amplified with the following primers, URA5 (5’ATGTCCTCCCAAGCCCTCGACTCCG3’) and SJ01 (5’TTAAGACC TCTGAACACCGTACTC3’). The genotypes were determined with a restriction fragment length polymorphism analysis (RFLP) of the *URA5* gene digested with restriction enzymes *HhaI* and *Sau96I* (Thermo Fisher Scientific, MA, USA) [3]. A set of standard laboratory reference strains representing each of the eight major molecular types were used for the molecular typing: WM148 (VNI), WM626 (VNII), WM 628 (VNIII), WM 629 (VNIV), WM 179 (VGI), WM 178 (VGII), WM 175 (VGIII), and WM 779 (VGIV) [3].

MLST analysis of the *C*. *neoformans/C*. *gattii* species complexes isolates was performed using the International Society for Human and Animal Mycology (ISHAM) consensus scheme of seven unlinked loci (*CAP59*, *GPD1*, IGS1, *LAC1*, *PLB1*, *SOD1*, and *URA5*). The allele types and sequence types (STs) were defined according to the ISHAM-MLST database for *C*. *neoformans* and *C*. *gattii* (http://mlst.mycologylab.org) [2]. All sequences are deposited in GenBank, and their accession numbers are described in S1 Table.

### Phylogenetic analysis

The generated sequences were manually edited and aligned with Clustal W using the program MEGA, version 6.06 (http://www.megasoftware.net) [27]. The concatenated alignments were then imported and analyzed using the neighbor-joining method with the *p*-distance. Bootstrap analysis, using 1,000 replicates with pairwise deletion, was employed to estimate the support for clades of the concatenate dataset.

### Genetic diversity analysis

The intra- and inter-population genetic variabilities were estimated by the number of polymorphic sites, number of haplotypes, haplotype diversity, nucleotide diversity, and average number of nucleotide differences. The number of polymorphic sites (S) is analogous to the number of alleles among sequences of genes [28]. The number of haplotypes (h) is a set of DNA variations, or polymorphisms, that tend to be inherited together, while haplotype diversity (Hd; also called gene diversity) is the probability of a difference between two randomly sampled alleles. Nucleotide diversity (π; also termed average pairwise difference) represents the average number of nucleotide differences (k) per site in pairwise comparisons of DNA sequences. To perform the analyses, isolates were stratified by different categories, including molecular types and source of isolations, and their DNA sequences were analyzed using DnaSP, version 6.12.01 (Universitat de Barcelona, Barcelona, Spain) [29, 30].

In addition, the selective neutrality of mutations was measured by Tajima’s D (D) tests for neutrality. This test distinguishes between the neutral and non-neutral evolution of a DNA sequence. The neutral evolution includes mutation-drift equilibrium, while the non-neutral evolution represents sequences evolving by directional or balancing selection, and demographic expansion or contraction. The Tajima’s D method (D) compares the average number and the estimated number of nucleotide differences from the number of segregating sites in the studied population [31]. Thus, the value of these tests would be close to zero under the neutrality. A negative or positive result suggests evidence of purifying (deleterious change) or balancing (the Darwinian or beneficial change) selection, respectively. *P*-values were generated using 1,000 simulations under a model of selective neutrality implemented in the DnaSP program [31].

### Linkage disequilibrium and recombination analysis

The presence of recombination within each population was performed using the percentage of phylogenetically compatible pairs of loci (PcP), and the index of association (*I*_*A*_), while the rBarD values of the different *C*. *neoformans/C*. *gattii* species complexes subpopulations were calculated using a clone-corrected dataset in order to avoid the bias of “high frequency” sequence types in the analysis in the software Multilocus version 1.3 using 1,000 randomizations. The absence of a difference between both datasets (*p* > 0.05) supports the null hypotheses of linkage equilibrium and sexual recombination, whereas significant differences supports linkage disequilibrium (LD) and clonality [32].The minimum number of recombination events (Rm) per gene and per population were calculated for each orthologous gene using the four-gamete test, which located pairs of the closest polymorphic sites within the 4 haplotypes likely to be generated by recombination between them; the DnaSP program was used [33]. The pairwise homoplasy index (PHI) test was used to infer if there was a statistical significance for a recombination by using SplitsTree, version 4.15.1 (http://www.splitstree.org) [34].

### Genetic differentiation based on allelic profile

A hierarchical analysis of molecular variance (AMOVA) was performed in GenAlEx 6.503 for Excel in order to examine the distribution of genetic variation, and determine the extent of connectivity among populations based on allelic profiles [35, 36]. AMOVA is a statistical technique that estimates the extent of genetic differentiation between individuals and populations directly from molecular data. The technique treats the raw molecular data as a pairwise matrix of genetic distances between all the possible combinations of isolates, with sub-matrices corresponding to the different hierarchical data-partitions (here, the genetic differences between different sources of isolation and geographical regions). In addition, the population differentiation test (*F*_*ST*_) from an AMOVA, assuming that the isolates were all haploids or homozygous diploids, was used to test the null hypotheses (H_0_) of no population differentiation. Values of *F*_*ST*_ can range from 0, which implies that the two populations are interbreeding freely (in these scenario we accept H_0_ and the *p* > 0.05), to 1, where all genetic variation is explained by the population structure and the two populations do not share any genetic diversity [37].

### *In vitro* analysis of virulence factors

*In vitro* analyses of important virulence factors of *C*. *neoformans/C*. *gattii* species complex were performed; these including the, growth rate at 37°C, melanin production, urease activity, and capsule formation. Each of these tests on the isolates was performed in triplicate.

### Growth rate at 37°C

The analysis of the cryptococcal growth dynamics based on cell numbers was performed according to previously published reports [38, 39], with minor modifications. Briefly, cryptococcal overnight cultures in a yeast-peptone-dextrose medium were washed twice and resuspended in fresh medium. The concentration of yeast cells was adjusted to optical density (OD) 600 at 0.1 in 10 ml of the medium. The culture was incubated at 37°C in a shaking incubator and the OD 600 was monitored at 0, 2, 4, 6, 8, 10, 12, and 24 h. The growth rate at 37°C was calculated by the population doubling time between the exponential and stationary growth phases.

### Melanin production

Each cryptococcal strain was grown in Sabouraud dextrose agar for 48 h at 30°C. Approximately 10^7^ yeast cells were suspended on phosphate-buffered saline before adding 10 ml of melanin induction medium (0.1% peptone, 0.2% dextrose and 10 mM dopamine hydrochloride) and incubated at 37°C in a shaking incubator. The initial cell concentration of each strain was determined by plate counts in duplicate. Supernatants of the culture were taken at 48 h, and their ODs at 475 nm were measured by spectrophotometer [40, 41].

### Urease activity

Each cryptococcal strain was grown in Sabouraud dextrose agar for 48 h at 30°C. The urease activity was determined according to a previous study, but with a minor modification [42]. Approximately 10^7^ yeast cells were suspended on phosphate-buffered saline and 50 μl of the suspension was added in each well of a 96-well plate containing a urea-broth base with a 2% urea solution (Thermo Fisher Scientific, Waltham, MA, USA). The plates were incubated at 37°C for 48 h and the ODs were measured at 550 nm to infer urease activity of each strain.

### Capsule formation

To investigate the capsule formation, stationary-phase fungal cultures were washed and resuspended in phosphate-buffered saline. Approximately 10^7^ yeast cells were then placed in 6-well plates containing 2 ml Dulbecco’s Modified Eagle Medium (DMEM) with 10% bovine serum albumin and they were incubated at 37°C with 5% CO_2_ for 48 h. Cells of each isolate were mounted in India ink to visualize the size of the polysaccharide capsule under a light microscope [15, 43]. A capsule size of at least 20 yeast cells per isolate was determined by the ImageJ program (NIH, Bethesda, MD, USA) and a calculation was made of the diameter ratio of capsule to capsule(from the cell wall to cell-wall boundary of each cell [15].

### Statistical analysis

Comparisons of the Hd data of different molecular types, or of the virulence characteristic data of different molecular types and sources, were performed with a two-tailed unpaired *t*-test using GraphPad Prism version 8.0.2 (GraphPad Software, California, USA). *P*-values of < 0.05 were considered statistically significant.

## Results

### Demographic data

Of the 228 isolates of the *C*. *neoformans*/*C*. *gattii* species complexes recovered during the pre-HIV-pandemic era (S1 Table), 204 (89.5%) and 24 (10.5%) strains were *C*. *neoformans* and *C*. *gattii*, respectively. Most isolates (186 strains; 81.5%) were recovered from clinical samples of 151 patients, followed by 35 environmental strains (15.4%) (isolated from animal dropping [74.29%], soil [14.29%], and tree hollows [5.71%]), and 7 veterinary strains (3.1%) (isolated from wound [57.14%], pus or exudate [28.57%], and animal lesions [14.29%]). In terms of their geographical distribution, 196 strains (86.0%) were recovered from the USA, followed by Thailand (14 strains; 6.1%), Denmark (10 strains; 4.4%), Italy (7 strains; 3.1%), and Canada (1 strain; 0.4%). The significant differences in genotype distributions between the pre- and during HIV pandemic eras based on geographic origin were shown in Table 1 [4, 44, 45].

**Table 1:** Comparison of genotype distributions of the pre- and during HIV pandemic eras based on geographic origin.

Molecular type	USA* (%)		Thailand* (%)		Denmark* (%)		Italy* (%)	
	Pre-HIV	During HIV	Pre-HIV	During HIV	Pre-HIV	During HIV	Pre-HIV	During HIV
**VNI**	131 (61.21%)	710 (73.05%)	13 (92.86%)	899 (95.84%)	2 (20%)	61 (58.65%)	0	231 (54.35%)
**VNII**	34 (15.89%)	5 (0.51%)	0	12 (1.28%)	0	5 (4.81%)	1 (11.11%)	0
**VNIII**	3 (1.40%)	55 (5.66%)	0	1 (0.11%)	0	13 (12.50%)	2 (22.22%)	79 (18.59%)
**VNIV**	24 (11.21%)	59 (6.07%)	0	1 (0.11%)	8 (80%)	21 (20.19%)	6 (66.67%)	112 (26.35%)
**VGI**	5 (2.34%)	6 (0.62%)	1 (7.14%)	4 (0.43%)	0	2 (1.92%)	0	3 (0.71%)
**VGII**	1 (0.47%)	108 (11.11%)	0	21 (2.24%)	0	2 (1.92%)	0	0
**VGIII**	16 (7.48%)	29 (2.98%)	0	0	0	0	0	0
**VGIV**	0	0	0	0	0	0	0	0
**Total**	214 (100%)	972 (100%)	14 (100%)	938 (100%)	10 (100%)	104 (100%)	9 (100%)	425 (100%)
**Reference**	This study	[44]	This study	[4, 44]	This study	[44, 45]	This study	[44]
*P*-value**	<0.0001		0.042		0.0001		<0.0001	

**Note**:

*Limited number of isolates and non-systematic strain collection

**Fisher’s exact test was performed by http://www.quantitativeskills.com/sisa/statistics/table2xr.htm

### The VNI/ST5, high-virulence genotype, was most prevalent among *C*. *neoformans*

**Fig 1:**
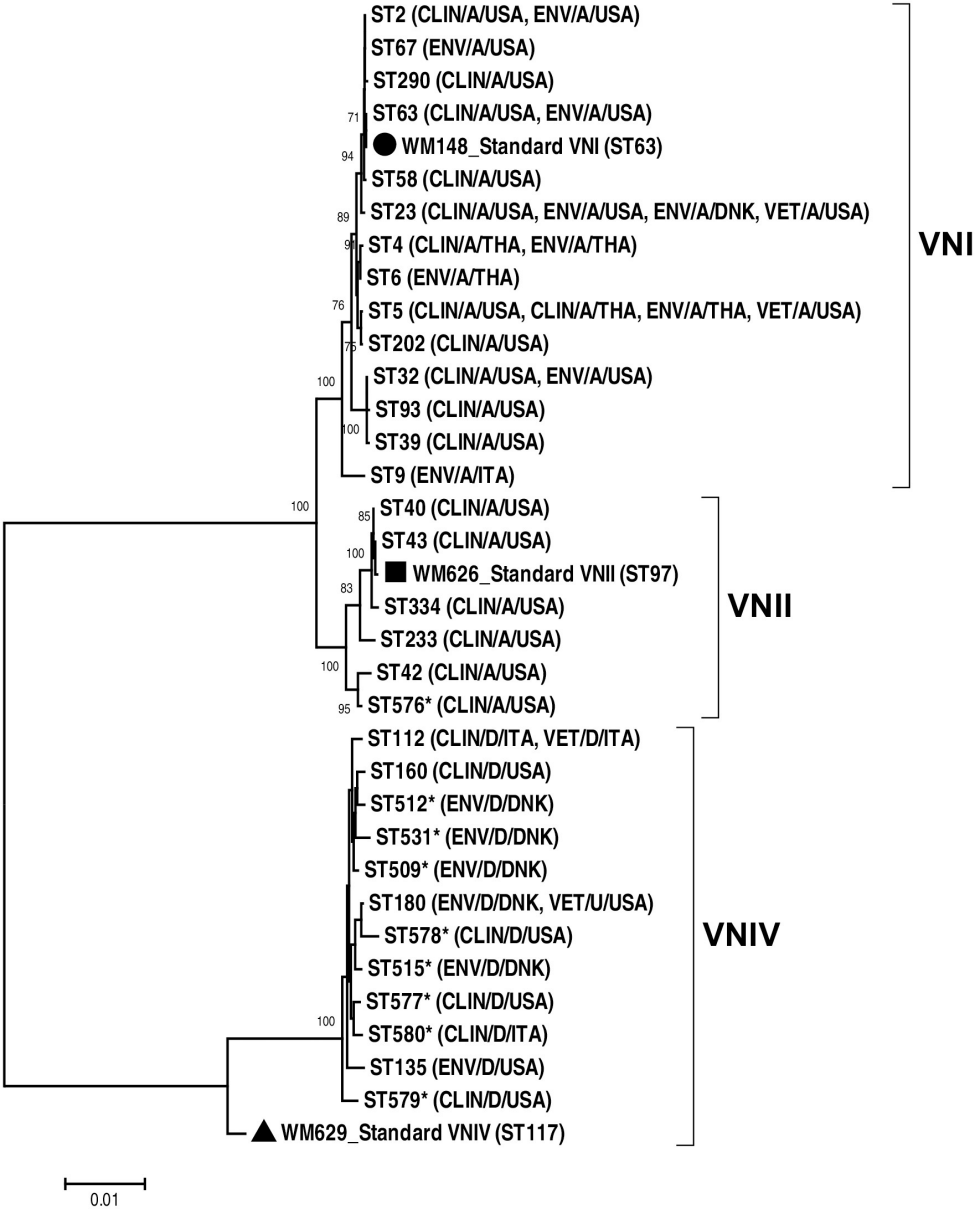
Neighbor-joining phylogenetic tree inferred using the concatenated sequences of the seven MLST loci (*CAP59*, *GPD1*, IGS1, *LAC1*, *PLB1*, *SOD1*, and the *URA5*) of the *C*. *neoformans* sequence types (STs) investigated in the present study. Bootstrap values, based on 1,000 replicates, are reported at each branch node and only bootstrap above 70% are presented in phylogenetic tree. (Abbreviations; CLIN: clinical, ENV: environmental, VET: veterinary, A: serotype A, D: serotype D, U: Unknown, USA: United State of America, THA: Thailand, DNK: Denmark, ITA: Italy, * represents a novel ST).

Overall, most isolates were identified as VNI (146 strains; 64.0%), followed by VNII (34 strains; 14.9%), VNIV (24 strains; 10.5%), VGIII (17 strains; 7.5%), VGI (6 strains; 2.6%), and VGII (1 strain; 0.5%). The most common allele type of the *CAP59*, *GPD1*, IGS1, *LAC1*, *PLB1*, *SOD1*, and *URA5* gene in *C*. *neoformans* was allele type AT1 (39.2%), AT1 (44.6%), AT1 (61.8%), AT5 (23.5%), AT1 (33.3%), AT1 (71.1%), and AT1 (42.6%), respectively (S1 Table). The MLST analysis divided the 204 *C*. *neoformans* isolates into 32 STs, comprised of ST5 (47 strains; 23.0%), ST2 (27 strains; 13.2%), ST63 (20 strains; 9.8%), ST40 (20 strains; 9.8%), and other STs (90 strains; 44.1%). Nine novel STs were identified in this study, specifically, ST576, ST509, ST512, ST515, ST531, ST577, ST578, ST579, and ST580. Most isolates were mating type alpha (197 strains, 96.9%) and 7 strains (3.4%) were mating type **a**, which all belonged to VNIV (S1 Table and Fig 1). When considering only the representative strains from each patient/source, the ST5 genotype was the most prevalent (42/170 strains; 24.7%; Table 2).

**Table 2:** Sequence type frequency of representative *C*. *neoformans/C*. *gattii* species complexes isolates.

Species	Molecular type	Sequence type^a^	Number of isolates			
			CLIN	ENV	VET	Total (%)
*C*. *neoformans*	VNI	2	23	3		26 (15.3%)
		4	1	8		9 (5.3%)
		5	38	2	2	42 (24.7%)
		6		1		1 (0.6%)
		23	10	5	1	16 (9.4%)
		32	7	3		10 (5.9%)
		39	2			2 (1.2%)
		58	1			1 (0.6%)
		63	11	1		12 (7.1%)
		67		1		1 (0.6%)
		93	4			4 (2.4%)
		202	1			1 (0.6%)
		290	1			1 (0.6%)
	VNII	9		1		1 (0.6%)
		40	10			10 (5.9%)
		42	4			4 (2.4%)
		43	4			4 (2.4%)
		233	1			1 (0.6%)
		334	1			1 (0.6%)
		**576**	1			1 (0.6%)
	VNIV	112	4		1	5 (2.9%)
		135		1		1 (0.6%)
		160	1			1 (0.6%)
		180		4	1	5 (2.9%)
		**509**		1		1 (0.6%)
		**512**		1		1 (0.6%)
		**515**		1		1 (0.6%)
		**531**		1		1 (0.6%)
		**577**	2			2 (1.2%)
		**578**	2			2 (1.2%)
		**579**	1			1 (0.6%)
		**580**	1			1 (0.6%)
**Total**			**131**	**34**	**5**	**170 (100.0%)**
*C*. *gattii*	VGI	51	1		1	2 (8.7%)
		106	1			1 (4.3%)
		162	1			1 (4.3%)
		208	1			1 (4.3%)
	VGII	20 (VGIIb)	1			1 (4.3%)
	VGIII	68 (VGIIIb)	2			2 (8.7%)
		75 (VGIIIa)	5		1	6 (26.1%)
		84 (VGIIIb)	1			1 (4.3%)
		86 (VGIIIb)	1			1 (4.3%)
		89 (VGIIIa)	1			1 (4.3%)
		93 (VGIIIa)	1			1 (4.3%)
		142 (VGIIIb)	2			2 (8.7%)
		146 (VGIIIa)	1			1 (4.3%)
		164 (VGIIIb)	1			1 (4.3%)
		209 (VGIIIb)	1			1 (4.3%)
**Total**			**21**		**2**	**23 (100.0%)**

**Abbreviations**: CLIN, clinical; ENV, environment; VET, veterinary

^*a*^**Bold with underline** signifies the novel sequence types found in this study

### High-virulence VGIIIa/serotype B clade was most prevalent among *C*. *gattii*

**Fig 2:**
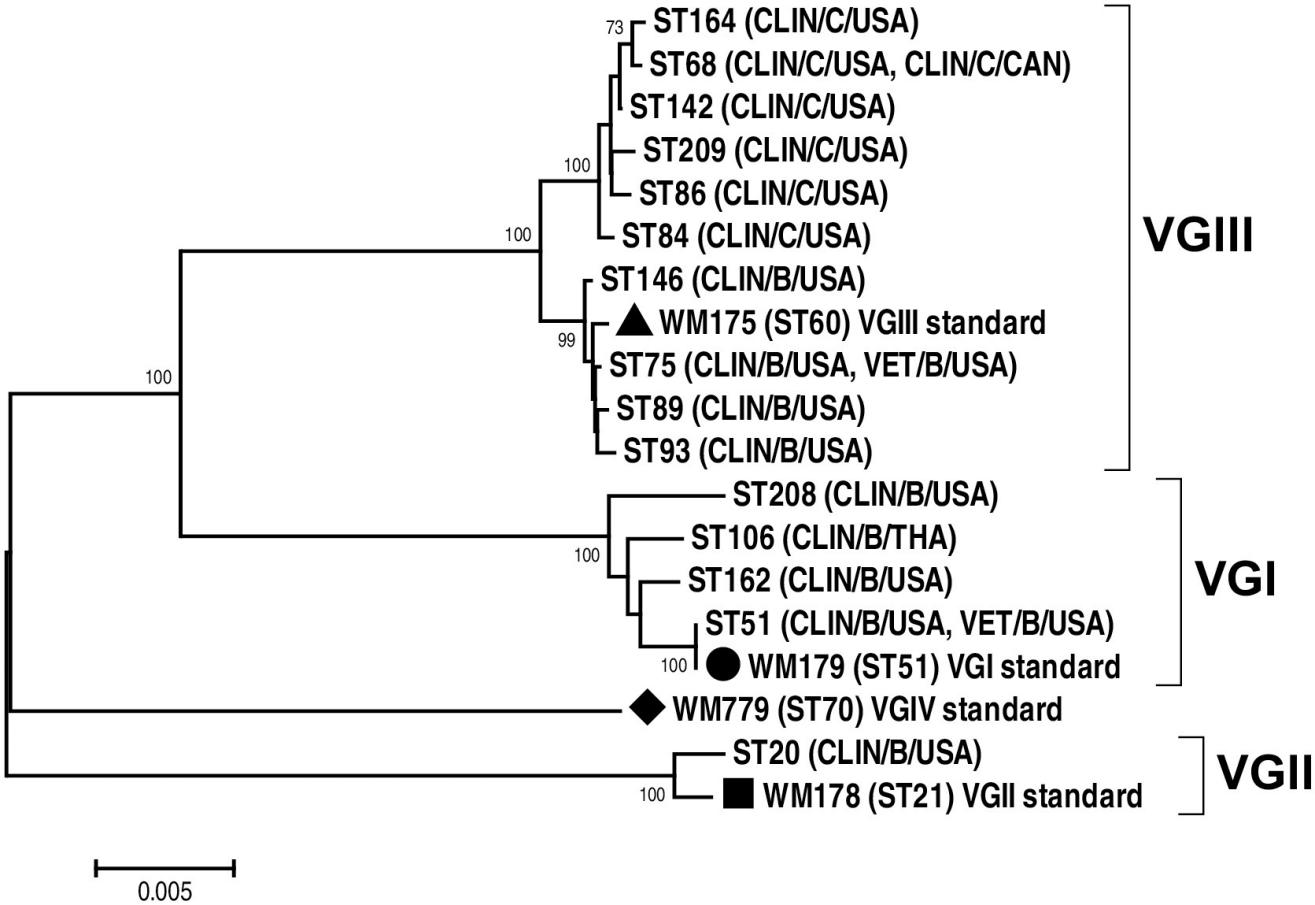
Neighbor-joining phylogenetic tree inferred using the concatenated sequences of the seven MLST loci (*CAP59*, *GPD1*, IGS1, *LAC1*, *PLB1*, *SOD1*, and the *URA5*) of the *C*. *gattii* sequence types (STs) investigated in the present study. Bootstrap values, based on 1,000 replicates, are reported at each branch node and only bootstrap above 70% are presented in phylogenetic tree. (Abbreviations; CLIN: clinical, VET: veterinary, B: serotype B, C: serotype C, USA: United State of America, THA: Thailand, CAN: Canada).

For *C*. *gattii*, the most common allele type of the *CAP59*, *GPD1*, IGS1, *LAC1*, *PLB1*, *SOD1*, and *URA5* genes was allele type AT18 (37.5%), AT9 (37.5%), AT1 (37.5%), AT3 (29.2%), AT6 (29.2%), AT28 (37.5%), and AT19 (29.2%), respectively (S1 Table). Of the 24 *C*. *gattii* isolates, 15 STs were identified: VGIII/ST75 (6 strains; 25.0%), VGIII/ST142 (2 strains; 8.3%), VGI/ST208 (2 strains; 8.3%), and other STs (14 strains; 58.4%). Most isolates were mating type alpha (19 strains, 79.2%), while 5 strains (20.8%) were mating type **a**, one in VGI and four in VGIII (S1 Table and Fig 2). When considering only the representative strains from each patient/source, the high-virulence VGIIIa/serotype B clade was the most common (9/23 isolates; 39.1% of all *C*. *gattii* isolates). The high-virulence VGIIa/ST20 genotype was the only one isolated, and the less virulent VGIIb/ST7 genotype was not present among the herein studied pre-HIV pandemic isolates (Table 2).

### VNIV had the highest genetic diversity among the *C*. *neoformans* species complex

Significant differences in the Hd values were observed in comparisons between the isolates of each molecular type (VNI *vs* VNIV, *p* = 0.040 and VNII *vs* VNIV, *p* = 0.003). VNIV isolates demonstrated the highest genetic diversity, having the highest Hd value (0.926) and π (0.00308; Table 3).

**Table 3:** Polymorphism summary and neutrality test for groups of isolates of *C*. *neoformans* species complex according to molecular types and sources of isolation.

	Locus	pb	S	h	Hd	k	π	D
VNI (n = 126)	*CAP59*	560	1	2	0.498	0.498	0.00089	1.860
	*GPD1*	538	2	4	0.526	13.586	0.02525	-2.456*
	IGS1	721	11	3	0.236	2.353	0.00326	0.404
	*LAC1*	470	5	5	0.745	1.691	0.00360	1.708
	*PLB1*	533	2	3	0.610	0.989	0.00185	2.471*
	*SOD1*	536	1	2	0.016	0.016	0.00003	-1.000
	*URA5*	637	2	3	0.549	0.605	0.00095	0.941
	Concatenated	4,001	24	13	0.815	6.659	0.00166	1.457
VNII (n = 21)	*CAP59*	560	1	2	0.381	0.381	0.00068	0.656
	*GPD1*	544	1	2	0.381	0.381	0.00070	0.656
	IGS1	720	21	4	0.471	3.371	0.00468	-1.594
	*LAC1*	471	3	2	0.095	0.286	0.00061	-1.727
	*PLB1*	533	3	2	0.381	1.143	0.00214	0.973
	*SOD1*	529	6	3	0.610	2.229	0.00421	1.056
	*URA5*	637	4	3	0.452	1.238	0.00194	0.324
	Concatenated	3,994	38	6	0.729	8.933	0.00224	-0.606
VNIV (n = 22)	*CAP59*	560	5	5	0.616	1.353	0.00242	-0.123
	*GPD1*	546	4	4	0.574	1.174	0.00215	0.119
	IGS1	685	9	9	0.858	3.174	0.00463	0.863
	*LAC1*	473	12	8	0.874	2.763	0.00584	-0.657
	*PLB1*	517	2	3	0.668	0.816	0.00158	1.048
	*SOD1*	525	4	5	0.511	0.921	0.00175	-0.530
	*URA5*	639	9	7	0.837	2.421	0.00379	-0.157
	Concatenated	3,940	44	12	0.926	12.147	0.00308	-0.082
Clinical (n = 131)	*CAP59*	560	44	6	0.692	7.489	0.01337	-0.206
	*GPD1*	537	58	7	0.709	9.961	0.01855	-0.242
	IGS1	678	107	11	0.542	17.641	0.02602	-0.372
	*LAC1*	467	52	10	0.800	8.946	0.01916	-0.186
	*PLB1*	517	42	7	0.756	7.712	0.01492	0.010
	*SOD1*	518	69	8	0.423	11.692	0.02257	-0.316
	*URA5*	637	45	12	0.685	7.274	0.01142	-0.358
	Concatenated	3,913	417	23	0.866	70.307	0.01797	-0.289
Environment (n = 34)	*CAP59*	560	42	7	0.733	15.927	0.02844	1.991
	*GPD1*	537	55	7	0.560	21.160	0.03940	1.903
	IGS1	682	100	8	0.638	36.367	0.05332	1.825
	*LAC1*	467	54	11	0.820	19.642	0.04206	1.786
	*PLB1*	517	39	6	0.802	15.269	0.02953	2.164*
	*SOD1*	527	64	4	0.437	25.194	0.04781	2.251*
	*URA5*	636	40	8	0.832	14.214	0.02235	1.634
	Concatenated	3,926	393	14	0.889	146.540	0.03733	1.962
Veterinary (n = 5)	*CAP59*	560	40	3	0.833	26.333	0.04702	2.148
	*GPD1*	537	52	3	0.833	34.333	0.06394	2.191
	IGS1	683	90	3	0.700	53.000	0.07760	1.723
	*LAC1*	468	42	4	0.900	24.600	0.05256	1.658
	*PLB1*	517	38	4	0.900	22.400	0.04333	1.714
	*SOD1*	527	63	2	0.600	37.800	0.07173	1.892*
	*URA5*	637	35	4	0.900	20.200	0.03171	1.518
	Concatenated	3,929	362	4	0.900	213.600	0.05436	1.726

**Abbreviations**: pb, total number of sites in alignments—excluding indels and missing data; S, polymorphic sites; h, number of haplotypes; Hd, haplotype diversity; k, average number of nucleotide difference; π, nucleotide diversity; D, Tajima’s D

*P-value < 0.05 as the significance level

The independent MLST loci analysis for the Hd of each molecular type showed that the *LAC1* locus was the most variable in the isolates of VNI (Hd = 0.745) and VNIV (Hd = 0.874), while least variable in the VNII (Hd = 0.095) isolates. The *SOD1* locus was the most variable in the VNII (Hd = 0.610) isolates, whereas it was the least variable in the isolates of VNI (Hd = 0.016) and VNIV (Hd = 0.511; Table 3). The neutrality test, Tajima’s D (D), showed evidence of balancing selection or expansion of rare polymorphisms for most loci for the overall *C*. *neoformans* species complex (*p* > 0.05; Table 3).

### Lowest genetic diversity was found among the clinical isolates of the *C*. *neoformans* species complex

When isolates were stratified according to their isolation sources, the Hd value was lowest among the clinical isolates (0.866) compared to those from other sources (ENV: 0.889, VET: 0.900), especially with those isolates from a veterinary source (CLIN *vs* VET, *p*-value = 0.041). The π value was lowest among the clinical isolates (0.01797) compared to the environment (0.03733) and veterinary (0.05436) isolates (CLIN *vs* ENV, *p* = 0.001; CLIN *vs* VET, *p* < 0.001; ENV *vs* VET, *p* = 0.035; Table 3).

When each locus was considered individually, the most variable one in the isolates from clinical, environmental, and veterinary source was *LAC1* (Hd = 0.800; π = 0.01916), *LAC1* (Hd = 0.820; π = 0.04206), and *LAC1*/*PLB1/URA5* (Hd = 0.900; π = 0.05256/0.04333/0.03171), respectively (Table 3). On the other hand, the least variable locus in the isolates from the clinical, environmental, and veterinary sources was *SOD1* with Hd = 0.423; π = 0.02257, Hd = 0.437; π = 0.04781, and Hd = 0.600; π = 0.07173, respectively (Table 3). No significant difference in neutrality test among each source of isolation was observed (*p* > 0.05; Table 3).

### The nucleotide diversity was higher among the VGI than VGIII isolates in *C*. *gattii*

The VGI and VGIII isolates showed no significant differences in their Hd values (0.867 and 0.875, respectively; *p* = 0.110), and the π values of the VGI isolates were not significantly different from those of the VGIII isolates (0.01436 and 0.00328, respectively; *p* = 0.305). No group was found to be under significant selective pressure according to the neutrality test (*p* > 0.05; Table 4). A comparison of the different sources among the *C*. *gattii* isolates was not pertinent as there were no environmental isolates available prior to HIV epidemic [46] and the number of veterinary isolates was too low (2 isolates; Table 4).

**Table 4:** Polymorphism summary and neutrality test for groups of isolates of *C*. *gattii* species complex according to molecular types and sources of isolation.

	Locus	pb	S	h	Hd	k	π	D
VGI (n = 5)	*CAP59*	557	5	3	0.733	2.267	0.00407	0.197
	*GPD1*	547	5	3	0.800	2.667	0.00488	1.219
	IGS1	690	6	4	0.867	2.867	0.00415	0.520
	*LAC1*	473	7	3	0.733	3.733	0.00789	1.267
	*PLB1*	532	2	3	0.733	0.867	0.00163	-0.050
	*SOD1*	701	10	4	0.867	4.333	0.00618	-0.063
	*URA5*	638	7	4	0.867	2.933	0.00460	-0.251
	Concatenated	4,133	158	4	0.867	59.333	0.01436	-0.924
VGIII (n = 17)	*CAP59*	557	5	2	0.529	2.647	0.00475	2.548*
	*GPD1*	547	4	4	0.669	1.471	0.00269	0.743
	IGS1	692	4	4	0.654	1.721	0.00249	1.390
	*LAC1*	472	10	6	0.787	3.956	0.00838	1.235
	*PLB1*	535	4	4	0.676	1.397	0.00261	0.553
	*SOD1*	711	2	3	0.581	0.632	0.00089	0.172
	*URA5*	638	6	6	0.779	1.794	0.00281	0.037
	Concatenated	4,152	35	10	0.875	13.618	0.00328	1.296

**Abbreviations**: pb, total number of sites in alignments—excluding indels and missing data; S, polymorphic sites; h, number of haplotypes; Hd, haplotype diversity; k, average number of nucleotide difference; π, nucleotide diversity, D; Tajima’s D

*P-value < 0.05 as the significance level

An independent analysis of each locus showed the least variability in the *CAP59*, *LAC1*, and *PLB1* loci in VGI (Hd = 0.733; π = 0.00407, 0.00789, and 0.00163, respectively) and in the *CAP59* locus in the VGIII group (Hd = 0.529; π = 0.00475). The most variable loci in the VGI isolates were the IGS1, *SOD1*, and *URA5* loci (Hd = 0.867; π = 0.00415, 0.00618, and 0.00460, respectively), while the *URA5* locus was the most variable of the VGIII isolates (Hd = 0.779; π = 0.00281;Table 4).

### Linkage disequilibrium were detected among *C*. *neoformans/C*. *gattii* species complexes populations

Among the different molecular types of the *C*. *neoformans* species complex, all molecular types values for PcP, *I*_*A*_, and rBarD were strongly rejected (*p* < 0.05) the null hypothesis of linkage equilibrium and free recombination (Table 5). The minimal number of recombination events (Rm) showed that the VNIV group had a higher Rm (10) than the VNI (2) and VNII groups (5), and the VNII and VNIV groups showed evidence for recombination (*p*-values for PHI test of < 0.001 and 0.019, respectively; Table 5). The independent loci analysis showed that two recombination events were identified in the IGS1 gene in the VNII population, one in the *CAP59*, *LAC 1*, and *URA5* genes, and three in the IGS1 gene of the VNIV population (Table 5).

**Table 5:** Multilocus linkage disequilibrium and recombination analyses amongst *C*.*neoformans/C*. *gattii* species complexes according to different molecular type and source of isolate.

Population	PcP^a^	*I*_*A*_^b^	rBarD^c^	Rm^d^	Rm per gene	PHI test
**Molecular type**						
VNI (n = 126)	0.809***	1.699***	0.307***	2		0.617
VNII (n = 21)	1.000***	4.078***	0.692***	5	IGS1 = 2	< 0.001
VNIV (n = 22)	0.714***	1.551***	0.267***	10	*CAP59*, *LAC 1*, *URA5* = 1/ IGS1 = 3	0.019
VGI (n = 5)	1.000*	3.854***	0.653***	4		0.568
VGIII (n = 17)	0.952***	3.375***	0.566***	4		0.094
**Sources of *C*. *neoformans* species complex**						
Clinical (n = 131)	0.809***	3.361***	0.566***	12	*URA5* = 1/ IGS1, *SOD1* = 2/ *LAC1* = 4	< 0.001
Environmental (n = 34)	0.952***	3.033***	0.521***	13	*GPD1*, IGS1 = 2/ *CAP59*, *LAC1* = 3	0.509
Veterinary (n = 5)	1.000_ns_	0.615_ns_	0.632_ns_	0		> 0.999
**Sources of *C*. *gattii* species complex**						
Clinical (n = 21)	0.952***	3.577***	0.605***	11	*URA5* = 1/ IGS = 2/ *LAC1*, *SOD* = 3	0.016
Veterinary (n = 2)^e^	ND	ND	ND	ND	ND	ND

^*a*^percentage of phylogenetically compatible pairs (PcP) of loci;

^*b*^index of association;

^*c*^scaled index of association (*I*_*A*_) by the number of loci (*m*– 1);

^*d*^minimal number of recombination based on each population;

^*e*^not determined—there were not enough samples for analysis

As to the *C*. *gattii* species complex, all molecular types showed values for PcP, *I*_*A*_, and rBarD that rejected (*p* < 0.05) the null hypothesis of linkage equilibrium and free recombination (Table 5). The PHI test did not show evidence of recombination among the molecular types of the *C*. *gattii* species complex, VGI (*p* = 0.568) and VGIII (*p* = 0.094; Table 5).

Based on the sources of the isolates, the *C*. *neoformans* species complex showed values for PcP, *I*_*A*_ and, rBarD that strongly rejected (*p* < 0.05) the null hypothesis of linkage equilibrium and free recombination for the clinical and environmental sources, but, this hypothesis was not rejected for the veterinary population (Table 5). The environmental (13) and clinical (12) isolates had a higher Rm than the veterinary isolates (0). Interestingly, the concatenated dataset showed evidence for recombination only in the clinical isolates (*p*-value of the PHI test < 0.001; Table 5). Taken together, the results from the different sources showed that, in the case of the clinical population, there was one recombination event in the *URA5* gene, two each in the IGS1 and *SOD1* genes, and four in the *LAC1* gene. As to the environmental population, there was two recombination events each in the *GPD1* and IGS1 genes, and three each in the *CAP59* and *LAC1* genes (Table 5).

The *C*. *gattii* species complex showed values for PcP, *I*_*A*_ and rBarD that rejected (*p* < 0.05) the null hypothesis of linkage equilibrium and free recombination (Table 5). The minimal number of recombination events (Rm) revealed that the clinical group had high Rm (10) and showed evidence for recombination (*p*-value for the PHI test of 0.016; Table 5). The independent loci analysis showed that in the case of the clinical population, there was one recombination event in the *URA5* gene, two in the IGS1 gene, and three each in the *LAC 1* and *SOD1* genes (Table 5).

### Genetic differentiation among *C*. *neoformans* species complex

Among the different genotype, AMOVA showed that the proportion of variance components within populations of the *C*. *neoformans* (51%) and *C*. *gattii* (67%) species complexes were higher than the proportions of variance components found among populations. The pairwise *F*_*ST*_ tests showed that both species complexes had significantly distinct subpopulations (*p* < 0.001) (Table 6).

**Table 6:** Hierarchical analysis of molecular variance (AMOVA) of different populations of *C*. *neoformans/C*. *gattii* species complexes.

	d.f.^a^	Sum of squares	Variance components	Percentage of variations	*F*_*ST*_^b^
**All *C*. *neoformans* isolates: VNI (126), VNII (21), and VNIV (22)**					
Among populations	2	220.928	1.660	49%	0.493 (*p* < 0.001)
Within populations	307	518.311	1.705	51%	
Total	309	739.239	3.365	100%	
**All *C*. *gattii* isolates: clinical (22) and veterinary (2)**					
Among populations	1	19.748	1.164	33%	0.332 (*p* < 0.001)
Within populations	40	88.824	2.337	67%	
Total	41	108.571	3.501	100%	
**All *C*. *neoformans* isolates: clinical (131), environmental (34), and veterinary (5)**					
Among populations	2	24.258	0.147	6%	0.058 (*p* < 0.001)
Within populations	405	971.408	2.399	94%	
Total	407	995.667	2.546	100%	
**All *C*. *gattii* isolates: clinical (22) and veterinary (2)**					
Among populations	1	4.189	0.190	6%	0.063 (*p* = 0.172)
Within populations	46	128.727	2.798	94%	
Total	47	132.917	2.988	100%	

^*a*^degrees of freedom;

^*b*^ the population differentiation test

Based on the sources of isolation, AMOVA showed that almost all variance components were found within populations rather than among populations in both the *C*. *neoformans* species complex (94%) and *C*. *gattii* species complex (94%). The pairwise *F*_*ST*_ tests were calculated among populations, and they showed that the *C*. *neoformans* species complex had significantly distinct populations (*p* < 0.001); in contrast, the *C*. *gattii* species complex did not have statistical support for differentiation (*p* = 0.172; Table 6).

### Sequential clinical strains showed less evidence of sequence type change

Of the 16 patients with sequential strains (S3 Table), 11 patients (68.8%) were identified as VNI, 3 patients (18.8%) as VNII, 1 patient (6.2%) as VNIV, and 1 patient (6.2%) as VGI. Majority of patients were infected by mating type alpha (15/16 patients, 93.8%) and one patient was infected by mating type **a**.

When each patient was considered individually, 15 patients (93.8%) were infected with the same sequence type including ST2/VNI (1 patient), ST5/VNI (3 patients), ST40/VNII (1 patient), ST42/VNII (1 patient), ST43/VNII (1 patient), ST58/VNI (1 patient), ST63/VNI (3 patients), ST93/VNI (1 patient), ST290/VNI (1 patient), ST578/VNIV (1 patient), and ST208/VGI (1 patient). One patient (patient B) was infected with a different sequence type: ST32/VNI and ST2/VNI (isolated after long-term treatment with a relapsed infection).

### High-virulence genotypes did not express more virulence factors than low-virulence genotypes

The values of the *in vitro* expression of the virulence factors among *C*. *neoformans/C*. *gattii* are presented in S2 Table. Based on the sequence type groups, the ST5 strains—recognized as a high prevalence and virulence genotype in the VNI molecular type [47, 48]—produced significantly smaller capsules than the strains in other STs in VNI (2.04 and 2.26, respectively; *p* = 0.001). Furthermore, there was no difference in the *in vitro* expressions of the virulence factors of the isolates of VNI and the other molecular types in *C*. *neoformans* (Table 7).

**Table 7:** Comparison of *in vitro* virulence characteristics of *C*. *neoformans* in ST5 and non-ST5 strains in the VNI group.

Characteristics	Group	ST5 (n = 47)	Non-ST5 (n = 99)	*P-*value^a^
Mean ± SD O.D. value of urease activity		0.382 ± 0.13	0.380 ± 0.15	0.938
Mean ± SD O.D. value of melanin production		0.180 ± 0.02	0.178 ± 0.02	0.565
Mean ± SD of population doubling time		114.28 ± 12.65	113.40 ± 11.01	0.831
Mean ± SD ratio of capsule production		2.04 ± 0.36	2.26 ± 0.39	0.001

**Abbreviation**: O.D., optical density

^*a*^Analyzed associations of different sequence-type groups by two-tailed unpaired *t-*test, with a *p*-value of < 0.05 as significance level

Likewise, there was no significant difference in the *in vitro* expressions of the virulence factors of VGIIIa/serotype B and VGIIIb/serotype C (Table 8).

**Table 8:** Comparison of *in vitro* virulence characteristics of *C*. *gattii* in VGIIIa/serotype B and VGIIIb/serotype C strains.

Characteristics	Group	VGIIIa/serotype B (n = 9)	VGIIIb/serotype C (n = 8)	*P-*value^a^
Mean ± SD O.D. value of urease activity		0.316 ± 0.12	0.281 ± 0.12	0.552
Mean ± SD O.D. value of melanin production		0.183 ± 0.02	0.184 ± 0.01	0.931
Mean ± SD of population doubling time		137.90 ± 26.17	136.90 ± 22.03	0.934
Mean ± SD ratio of capsule production		2.88 ± 0.36	2.99 ± 0.39	0.685

**Abbreviation**: O.D., optical density

^*a*^Analyzed associations of different sequence-type groups by two-tailed unpaired *t*-test, with a *p*-value of < 0.05 as significance level

## Discussion

Before the standard molecular typing system had been established for the *C*. *neoformans*/*C*. *gattii* species complexes by Meyer et al in 1999 [49], our understanding of the cryptococcal molecular epidemiology was extremely limited. Since then, significant progress has been made in molecular typing as well as in our understanding of the genotype-specific characteristics of the two species complexes [13, 15, 22, 38, 50]. Still, the association of cryptococci with immunocompromised humans became clear only after the advent of the HIV-pandemic in 1980s. Since the populations of the two species complexes prior to the HIV pandemic has not been studied, we conducted a molecular epidemiological study of the isolates from the pre-HIV era and analyzed the *in vitro* expressions of the known virulence factors. As is the case with the isolates of the post-HIV era, the most prevalent molecular type of *C*. *neoformans* was VNI (146/204 isolates; 71.6%), indicating that VNI is a major molecular type of *C*. *neoformans* in both the pre-HIV- and HIV-pandemic eras (S4 Table). Interestingly, we found that VGIII was the predominant molecular type of the *C*. *gattii* species complex in our collection during the pre-HIV-pandemic era (17/24 isolates; 70.8%). This finding contrasts with the fact that the VGI and VGII molecular types are currently known as the most frequent types among the *C*. *gattii* species complex [44, 51]. This might be due to the geographic distribution that VGIII was commonly reported in the USA whereas the isolates were mostly recovered in the USA (Table 1 and S4 Table) [22, 51].

Further genotype analysis of *C*. *neoformans* by MLST showed that ST5/VNI was the most common genotype (43/164 isolates; 26.2%) among the clinical isolates of *C*. *neoformans*. This result is similar to the findings of a recent systematic review of Asian cryptococcosis in which ST5/VNI was reported to be the most common genotype among non-HIV patients in both East Asian (87.9%) and other Asian (39.3%) countries [52]. Our findings is also similar to what has been reported for Vietnam (83.7%) [47] and Laos (25%) [53]. Three other common genotypes—namely, ST2/VNI (13.2%), ST63/VNI (9.8%), and ST40/VNII (9.8%)—were reported in Asia, Africa, Europe, and the United States [4, 10, 11, 23, 54]. Interestingly, the most common genotype among the environmental isolates, ST4/VNI (25%), was also the most common (32.6%) sequence type isolated from HIV patients outside of the East Asian countries [52]. This result concurs with the proposal that HIV patients contracted cryptococcosis from their environment as the diversity of the genotypes in the environment is reflected in the clinical isolates [55]. Similarly, ST4/VNI has been found to be very rare among clinical isolates from Europe, the Mediterranean area (1%) [54], and Brazil (0%) [56]; predictably, they have rarely been isolated from the environment in those geographic areas.

Among the isolates from both clinical and environmental sources, 9 novel STs were identified—one in VNII (ST576), and 8 in VNIV (ST509, ST512, ST515, ST531, ST577, ST578, ST579, and ST580. This suggests, that these STs may have been suppressed during the post-pandemic era. As the response to stressful conditions, including the host immune response, were different for each serotype, survival of these 9 STs may have been affected during the post-HIV era since HIV patients are the major source for cryptococcal isolates [57–59]. On the other hand, this might simply have been caused by a sampling bias during the HIV pandemic. Therefore, further sampling of more isolates during this present time would be beneficial.

Among the *C*. *gattii* strains, the ST75/VGIII (6/24 strains; 25.0%) genotype was the most common ST isolated during the pre-HIV-pandemic era. The prevalence of this ST is actually consistent with the prevalence of ST isolated during the HIV-pandemic era as ST75/VGIII was the most common causative agent of human and animal cryptococcosis due to *C*. *gattii* in North and South America [20, 22, 60]. Moreover, a recent study reported that the VGIIIa/serotype B was more virulent than VGIIIb/serotype C [18]. These data could support the predominance of this ST in North America. Surprisingly, the absence of the high-virulence Vancouver outbreak genotype, ST20/VGIIa during the pre-HIV-pandemic era supports the hypothesis that this genotype was a result of a recent *Cryptococcus* evolution/recombination [14, 61].

Previous population genetic analyses of HIV-pandemic *C*. *neoformans* isolates have shown that the VNIV isolates are genetically more diverse and have a higher recombination rate than the VNI and VNII isolates [9, 54, 62–64]. Our study showed the same results suggesting that the manner of VNIV propagation is different from those of VNI and VNII (primarily clonal expansion in VNI and VNII *vs* recombinational events in VNIV). This was confirmed by our result showing the highest Rm in VNIV. Moreover, we found that the clinical isolates of *C*. *neoformans* had a lower genetic diversity (Hd = 0.866, π = 0.01797) than the environmental isolates (Hd = 0.889, π = 0.03733). This finding is similar to that of a European study, in which a higher diversity was observed among the environmental isolates than the clinical isolates [54]. However, the contradiction between a high genetic diversity (Hd = 0.900, π = 0.05436) for the veterinary isolates remains disputable; more isolates should be investigated since only 5 were analyzed in the current study. As for the *C*. *gattii* species complex, the degree of genetic diversity between the different molecular types which was similar except for the higher π value was higher for VGI compared than VGIII (0.01436 *vs* 0.00328). This value could have been affected by the number of polymorphic sites because VGI had approximately 5 times as many polymorphic sites as VGIII (158 *vs* 35) [65]. Moreover, there has been a report of a low genetic flow in the VGIII molecular type [18].

The linkage equilibrium revealed significant disequilibrium and the genetic differentiation analysis detected a significantly low genetic differentiation in almost all subpopulations of the *C*. *neoformans/C*. *gattii* species complex with different molecular types and sources of isolation These finding suggest limited genetic exchange among each population, as proposed by previous studies [5, 9, 12, 66, 67]. This low genetic exchange or recombination rate might increase the risk of extinction due to reductions in the genetic diversity and a loss of population fitness, as suggested by a recent study [68]. Therefore, some populations in the pre-HIV pandemic strains might have been suppressed during the HIV pandemic, as in the cases of the novel pre-HIV pandemic genotypes.

As to the polymorphism of each locus of the housekeeping genes, the *LAC1* gene possessed the highest number of haplotypes, a higher π, and a higher Rm compared with those of the other genes in both *C*. *neoformans* species complex. In addition, the *LAC1* gene in environmental isolates had a higher π value than the clinical isolates for both VNI and VNIV; this can be explained by the fact that the fungus has to adapt itself to the substrate in order to survive in the environment [54]. In fact, a recent study on the *LAC1* gene showed that polymorphism within the *LAC1* gene, whose protein product catalyzes melanin synthesis, to be associated with variable melanin levels, suggesting a correlation between gene polymorphism and melanin production levels [69].

One of the 16 patients with sequential strains was infected with different cryptococcal strains. Though this has rarely occurred, mixed and/or sequential infections by different cryptococcal strains in a single patient have been reported [70–72]. One study reported that a patient was infected with two different strains after 11 days in hospital, and that the second strain prove to be less susceptible to antifungal treatment than the first [70]. Another study reported that 4 patients had relapsed infections caused by isolates that were genetically different from the initial etiologic agents [71]. Similar circumstances were observed in our case of sequential infections in which a relapsed infection caused by a different isolate after long-term treatment. In another study, some HIV patients in the Ivory Coast were suspected of having been infected by mixed strains, but only one strain was isolated at diagnosis; and the second strain, which was more resistant to antifungal drug treatment, emerged later in a fungal culture [73].

Although a higher virulence has been documented for the ST5/VNI and VGIIIa/serotype B [18, 47], our *in vitro* analysis of the virulence factors *in vitro* did not correlate with these strain types. There were no significant differences between the ST5/VNI and non-ST5/VNI strains, except that there was a smaller capsule production in the less virulent ST5/VNI strains. While the difference in capsule size was significant, it was minimal (2.04 *vs* 2.26); it is also known that the *in vitro* capsule size does not correlate with the *in vivo* virulence of *C*. *neoformans* [74]. Only the absence or presence of the capsule has been directly correlated with cryptococcal virulence [75–77], and this finding has been supported by pathogenesis studies that showed the capsule accumulation *in vivo* was higher than *in vitro* [78, 79]. A similar picture was seen with the *C*. *gattii in vitro* expression of virulence factors: no significant difference was observed between the high-virulence VGIIIa/serotype B and the low-virulence VGIIIb/serotype C strains.

In conclusion, these data marked high genetic variability and recombination events in the pre-HIV-pandemic strains of the *C*. *neoformans/C*. *gattii* species complexes. The identification of novel STs in this study suggests that some STs were either suppressed or disappeared during the HIV pandemic. The difference in the virulence of the high- and low-virulence genotypes might not have developed until after the start of the pandemic. However, to gain in-depth information on the evolution of these historical strains, analysis of amino acid changes and specific indels with a specifically targeted whole gene sequencing is needed. Moreover, due to a low number of *C*. *gattii* species complex strains used in the current study, additional *C*. *gattii* species complex strains need to be collected for use in further investigations.

## Supporting information

S1 Table MLST data of the 228 *C*. *neoformans/C*. *gattii* species complexes strains from the pre-HIV-pandemic era used in this study. (XLSX)

S2 Table *In vitro* virulence data of the 228 *C*. *neoformans/C*. *gattii* species complexes strains from the pre-HIV-pandemic era used in this study. (DOCX)

S3 Table MLST profile and clinical data of sequential strains from 16 different patients. (XLSX)

S4 Table Comparison of the genotype distributions of the pre- and during HIV pandemic eras for the clinical strains isolated from USA. (DOCX)
