# Supplementary material for: Population diversity and virulence characteristics of Cryptococcus neoformans/C. gattii species complexes isolated during the pre-HIV-pandemic era
Source: PLoS Negl Trop Dis. 2020 Oct 5;14(10):e0008651. doi: 10.1371/journal.pntd.0008651 (PMC7535028; doi:10.1371/journal.pntd.0008651)
Supplement: S2 Table — (DOCX) [file pntd.0008651.s002.docx]

**S2 Table** MLST profile of sequential clinical data from 16 different patients.

| **Patient** | **Isolate** | ***URA5*-RFLP** | **Mating type** | **Allelic profiles** | | | | | | | **ST** |
| --- | --- | --- | --- | --- | --- | --- | --- | --- | --- | --- | --- |
|  |  |  |  | ***CAP59*** | ***GPD1*** | **IGS1** | ***LAC1*** | ***PLB1*** | ***SOD1*** | ***URA5*** |  |
| A | 1 | VNI | alpha | 7 | 1 | 1 | 18 | 1 | 1 | 1 | 63 |
|  | 2 | VNI | alpha | 7 | 1 | 1 | 18 | 1 | 1 | 1 | 63 |
|  | 3 | VNI | alpha | 7 | 1 | 1 | 18 | 1 | 1 | 1 | 63 |
|  | 4 | VNI | alpha | 7 | 1 | 1 | 18 | 1 | 1 | 1 | 63 |
|  | 5 | VNI | alpha | 7 | 1 | 1 | 18 | 1 | 1 | 1 | 63 |
| **B** | **1** | **VNI** | **alpha** | **1** | **1** | **10** | **3** | **4** | **1** | **1** | **32** |
|  | **2** | **VNI** | **alpha** | **7** | **1** | **1** | **1** | **1** | **1** | **2** | **2** |
| C | 1 | VGI | alpha | 24 | 43 | 74 | 31 | 33 | 80 | 39 | 208 |
|  | 2 | VGI | alpha | 24 | 43 | 74 | 31 | 33 | 80 | 39 | 208 |
| D | 1 | VNIV | a | 14 | 21 | 42 | 23 | 14 | 17 | 34 | 578 |
|  | 2 | VNIV | a | 14 | 21 | 42 | 23 | 14 | 17 | 34 | 578 |
|  | 3 | VNIV | a | 14 | 21 | 42 | 23 | 14 | 17 | 34 | 578 |
| E | 1 | VNI | alpha | 1 | 3 | 1 | 5 | 2 | 1 | 1 | 5 |
|  | 2 | VNI | alpha | 1 | 3 | 1 | 5 | 2 | 1 | 1 | 5 |
| F | 1 | VNI | alpha | 7 | 1 | 1 | 18 | 1 | 1 | 1 | 63 |
|  | 2 | VNI | alpha | 7 | 1 | 1 | 18 | 1 | 1 | 1 | 63 |
| G | 1 | VNII | alpha | 8 | 10 | 15 | 8 | 12 | 3 | 11 | 42 |
|  | 2 | VNII | alpha | 8 | 10 | 15 | 8 | 12 | 3 | 11 | 42 |
| H | 1 | VNI | alpha | 1 | 1 | 1 | 18 | 1 | 1 | 2 | 58 |
|  | 2 | VNI | alpha | 1 | 1 | 1 | 18 | 1 | 1 | 2 | 58 |
| I | 1 | VNI | alpha | 7 | 1 | 1 | 1 | 1 | 1 | 2 | 2 |
|  | 2 | VNI | alpha | 7 | 1 | 1 | 1 | 1 | 1 | 2 | 2 |
| J | 1 | VNI | alpha | 1 | 3 | 1 | 5 | 2 | 1 | 1 | 5 |
|  | 2 | VNI | alpha | 1 | 3 | 1 | 5 | 2 | 1 | 1 | 5 |
| K | 1 | VNI | alpha | 1 | 23 | 10 | 3 | 4 | 1 | 1 | 93 |
|  | 2 | VNI | alpha | 1 | 23 | 10 | 3 | 4 | 1 | 1 | 93 |
|  | 3 | VNI | alpha | 1 | 23 | 10 | 3 | 4 | 1 | 1 | 93 |
|  | 4 | VNI | alpha | 1 | 23 | 10 | 3 | 4 | 1 | 1 | 93 |
| L | 1 | VNI | alpha | 7 | 1 | 1 | 18 | 1 | 35 | 2 | 290 |
|  | 2 | VNI | alpha | 7 | 1 | 1 | 18 | 1 | 35 | 2 | 290 |
| M | 1 | VNII | alpha | 2 | 9 | 14 | 8 | 11 | 12 | 4 | 40 |
|  | 2 | VNII | alpha | 2 | 9 | 14 | 8 | 11 | 12 | 4 | 40 |
|  | 3 | VNII | alpha | 2 | 9 | 14 | 8 | 11 | 12 | 4 | 40 |
|  | 4 | VNII | alpha | 2 | 9 | 14 | 8 | 11 | 12 | 4 | 40 |
|  | 5 | VNII | alpha | 2 | 9 | 14 | 8 | 11 | 12 | 4 | 40 |
|  | 6 | VNII | alpha | 2 | 9 | 14 | 8 | 11 | 12 | 4 | 40 |
|  | 7 | VNII | alpha | 2 | 9 | 14 | 8 | 11 | 12 | 4 | 40 |
|  | 8 | VNII | alpha | 2 | 9 | 14 | 8 | 11 | 12 | 4 | 40 |
|  | 9 | VNII | alpha | 2 | 9 | 14 | 8 | 11 | 12 | 4 | 40 |
|  | 10 | VNII | alpha | 2 | 9 | 14 | 8 | 11 | 12 | 4 | 40 |
|  | 11 | VNII | alpha | 2 | 9 | 14 | 8 | 11 | 12 | 4 | 40 |
| N | 1 | VNI | alpha | 1 | 3 | 1 | 5 | 2 | 1 | 1 | 5 |
|  | 2 | VNI | alpha | 1 | 3 | 1 | 5 | 2 | 1 | 1 | 5 |
|  | 3 | VNI | alpha | 1 | 3 | 1 | 5 | 2 | 1 | 1 | 5 |
|  | 4 | VNI | alpha | 1 | 3 | 1 | 5 | 2 | 1 | 1 | 5 |
| O | 1 | VNII | alpha | 2 | 9 | 14 | 8 | 11 | 11 | 4 | 43 |
|  | 2 | VNII | alpha | 2 | 9 | 14 | 8 | 11 | 11 | 4 | 43 |
| P | 1 | VNI | alpha | 7 | 1 | 1 | 18 | 1 | 1 | 1 | 63 |
|  | 2 | VNI | alpha | 7 | 1 | 1 | 18 | 1 | 1 | 1 | 63 |
|  | 3 | VNI | alpha | 7 | 1 | 1 | 18 | 1 | 1 | 1 | 63 |
|  | 4 | VNI | alpha | 7 | 1 | 1 | 18 | 1 | 1 | 1 | 63 |

**Abbreviation:** ST, sequence type
