# Supplementary material for: Population diversity and virulence characteristics of Cryptococcus neoformans/C. gattii species complexes isolated during the pre-HIV-pandemic era
Source: PLoS Negl Trop Dis. 2020 Oct 5;14(10):e0008651. doi: 10.1371/journal.pntd.0008651 (PMC7535028; doi:10.1371/journal.pntd.0008651)
Supplement: S4 Table — (DOCX) [file pntd.0008651.s004.docx]

**S4 Table** Comparison of genotype distributions of the pre- and during HIV pandemic eras among clinical strains isolated from USA

| Molecular type | USA* (%) | |
| --- | --- | --- |
|  | Pre-HIV | During HIV |
| VNI | 116  (64.44%) | 33  (37.08%) |
| VNII | 33  (18.33%) | 18  (20.22%) |
| VNIII | 3  (1.67%) | 1  (1.12%) |
| VNIV | 8  (4.44%) | 1  (1.12%) |
| VGI | 4  (2.22%) | 3  (3.37%) |
| VGII | 1  (0.56%) | 4  (4.49%) |
| VGIII | 15  (8.33%) | 29  (32.58%) |
| VGIV | 0 | 0 |
| Total | 180  (100%) | 89  (100%) |
| Reference | This study | [1-7] |
| *P* value** | <0.0001 | |

**Note:** *Limited number of isolates and non-systematic strain collection

**Fisher’s exact test was performed by

<http://www.quantitativeskills.com/sisa/statistics/table2xr.htm>

**References**

1. Litvintseva AP, Thakur R, Vilgalys R, Mitchell TG. Multilocus sequence typing reveals three genetic subpopulations of *Cryptococcus neoformans* var. *grubii* (serotype A), including a unique population in Botswana. Genetics. 2006;172(4):2223-38. Epub 2005/12/03. doi: 10.1534/genetics.105.046672. PubMed PMID: 16322524; PubMed Central PMCID: PMCPMC1456387.

2. Byrnes EJ, 3rd, Li W, Ren P, Lewit Y, Voelz K, Fraser JA, et al. A diverse population of *Cryptococcus gattii* molecular type VGIII in southern Californian HIV/AIDS patients. PLoS pathogens. 2011;7(9):e1002205. Epub 2011/09/13. doi: 10.1371/journal.ppat.1002205. PubMed PMID: 21909264; PubMed Central PMCID: PMCPMC3164645.

3. Litvintseva AP, Mitchell TG. Most environmental isolates of *Cryptococcus neoformans* var. *grubii* (serotype A) are not lethal for mice. Infection and immunity. 2009;77(8):3188-95. Epub 2009/06/03. doi: 10.1128/iai.00296-09. PubMed PMID: 19487475; PubMed Central PMCID: PMCPMC2715664.

4. Sellers B, Hall P, Cine-Gowdie S, Hays AL, Patel K, Lockhart SR, et al. *Cryptococcus gattii*: an emerging fungal pathogen in the Southeastern United States. Am J Med Sci. 2012;343(6):510-1. Epub 2012/02/09. doi: 10.1097/MAJ.0b013e3182464bc7. PubMed PMID: 22314106.

5. Walraven CJ, Gerstein W, Hardison SE, Wormley F, Lockhart SR, Harris JR, et al. Fatal disseminated *Cryptococcus gattii* infection in New Mexico. PloS one. 2011;6(12):e28625. Epub 2011/12/24. doi: 10.1371/journal.pone.0028625. PubMed PMID: 22194869; PubMed Central PMCID: PMCPMC3237461.

6. Byrnes EJ, 3rd, Li W, Lewit Y, Perfect JR, Carter DA, Cox GM, et al. First reported case of *Cryptococcus gattii* in the Southeastern USA: implications for travel-associated acquisition of an emerging pathogen. PloS one. 2009;4(6):e5851. Epub 2009/06/12. doi: 10.1371/journal.pone.0005851. PubMed PMID: 19516904; PubMed Central PMCID: PMCPMC2689935.

7. Upton A, Fraser JA, Kidd SE, Bretz C, Bartlett KH, Heitman J, et al. First contemporary case of human infection with *Cryptococcus gattii* in Puget Sound: evidence for spread of the Vancouver Island outbreak. Journal of clinical microbiology. 2007;45(9):3086-8. Epub 2007/06/29. doi: 10.1128/jcm.00593-07. PubMed PMID: 17596366; PubMed Central PMCID: PMCPMC2045307.
